# Jasmonic acid negatively regulates branch growth in pear

**DOI:** 10.3389/fpls.2023.1105521

**Published:** 2023-02-07

**Authors:** Yuanyuan Cheng, Chenglin Liang, Zhiyun Qiu, Siqi Zhou, Jianlong Liu, Yingjie Yang, Ran Wang, Jie Yin, Chunhui Ma, Zhenhua Cui, Jiankun Song, Dingli Li

**Affiliations:** ^1^ Qingdao Key Laboratory of Genetic Improvement and Breeding in Horticultural Plants, Engineering Laboratory of Genetic Improvement of Horticultural Crops of Shandong Province, College of Horticulture, Qingdao Agricultural University, Qingdao, China; ^2^ Haidu College, Qingdao Agricultural University, Laiyang, China

**Keywords:** pear, branch, JA, transcriptional regulation, less branching mutant plant

## Abstract

The quality of seedlings is an important factor for development of the pear industry. A strong seedling with few branches and suitable internodes is ideal material as a rootstock for grafting and breeding. Several branching mutants of pear rootstocks were identified previously. In the present study, ‘QAU-D03’ (*Pyrus communis* L.) and it’s mutants were used to explore the mechanism that affects branch formation by conducting phenotypic trait assessment, hormone content analysis, and transcriptome analysis. The mutant plant (MP) showed fewer branches, shorter 1-year-old shoots, and longer petiole length, compared to original plants (OP), i.e., wild type. Endogenous hormone analysis revealed that auxin, cytokinin, and jasmonic acid contents in the stem tips of MP were significantly higher than those of the original plants. In particular, the jasmonic acid content of the MP was 1.8 times higher than that of the original plants. Transcriptome analysis revealed that *PcCOI1*, which is a transcriptional regulatory gene downstream of the jasmonic acid signaling pathway, was expressed more highly in the MP than in the original plants, whereas the expression levels of *PcJAZ* and *PcMYC* were reduced in the MP compared with that of the original plants. In response to treatment with exogenous methyl jasmonate, the original plants phenotype was consistent with that of the MP in developing less branches. These results indicate that jasmonic acid negatively regulates branch growth of pear trees and that jasmonic acid downstream regulatory genes play a crucial role in regulating branching.

## 1 Introduction

Grafting is a common method for asexual propagation of plants and for raising fruit tree seedlings. Pear rootstock with a strong stem, few branches, and suitable internodes is ideal material for grafting and breeding. Branch development and growth are regulated by a complex network of factors involving plant growth and development, hormone regulation, transcriptional regulation, and other factors.

Bud mutation is an important means of causing changes in branching through plant somatic variation (Zhao et al., 2021). The meristem cells of buds undergo genetic changes during cell division, leading to the development of lateral branches (Leng et al., 2021). The shoot apical meristem (SAM) is also crucial to plant development and is responsible for the development of leaves, stems, and flowers (Han et al., 2019). The SAM gives rise to leaf primordia during the vegetative growth stage and an inflorescence meristem in the transitional stage from vegetative growth to reproductive growth. An axillary meristem (AM) gives rise to a lateral bud primordium, which develops into a lateral bud and ultimately forms lateral branches (Grbić and Bleecker, 2000; Tanaka et al., 2013; ). The development of plant meristems mainly depends on the population of multifunctional stem cells in plants, and is affected by hormones and environmental factors.

Plant growth and development adapt to environmental changes through hormone signals, which provide a biochemical connection between the environment and cellular responses (Mur et al., 2006; Clarke et al., 2009). The auxin and cytokinin regulatory pathways are the main factors that regulate meristem formation and differentiation (Depuydt and Hardtke, 2011). Auxin combines with brassinolide (BR) to induce *SMALL AUXIN UPREGULATED RNA 10 (SAUR10)*, thus participating in the regulation of branching angle (Bemer et al., 2017). Cytokinin is considered to be a second messenger that transmits auxin signals to lateral buds (Leyser, 2003). Cytokinin plays an important role in regulating leaf tip dominance and axillary bud growth. The expression level of *PsIPT*, a critical enzyme involved in cytokinin biosynthesis in pea, increases at the node after decapitation. Bud growth after decapitation is caused by the local accumulation of cytokinin (Tanaka et al., 2006). In poplar, overexpression of *GA2ox* leads to an increase in the number of tillers or branches, which indicates that gibberellin may play a negative role in the control of poplar bud branching (Zawaski and Busov, 2014). *RAX1* promotes early formation of the AM, negatively regulates the gibberellin content of the stem tip, and affects the timing of AM development (Fambrini et al., 2017; Nie et al., 2018). Jasmonic acid is involved in regulating responses to biological and abiotic stresses, as well as growth and development of plants (Wasternack and Hause, 2013; Campos et al., 2014). In the jasmonic acid signaling pathway, *COI1*, as a downstream regulatory element of jasmonic acid, participates in almost all JAs regulatory processes (Ye et al., 2012). The *COI1* gene encodes a F-box protein, which is a component of E3 ubiquitin ligase (Xie et al., 1998). The enzyme is associated with meristem arrest and apical dominance (Zhai et al., 2015). The *coi1* mutant shows strong apical dominance and enhanced meristem longevity (Kim et al., 2013). J*asmonate ZIM-domain (JAZ)* proteins play an inhibitory role in the JA signaling pathway (Chini et al., 2007; Thines et al., 2007; Yan et al., 2007). Overexpression of *JAZ* can cause increase in the number of lateral branches in tomato (Yu et al., 2018).

The regulation of transcription is important in the control of eukaryotic gene expression. Transcription factors are involved in all aspects of plant growth and development. The *SQUAMOSA PROMOTER BINDING PROTEIN-LIKE 13 (SPL13)* gene encodes a *SBP* transcription factor, which is mainly expressed in meristems and is critical to regulating the branching and vegetative growth of alfalfa plants (Gao et al., 2018). Overexpression of *SPL13* inhibits the growth of axillary buds and reduces the number of lateral branches. *MsMYB112* RNA interference promotes branching, which indicates that *MYB112* inhibits the growth of lateral branches in alfalfa (Stracke et al., 2001; Gao et al., 2018; ). In addition, *TCP* transcription factors are involved in lateral meristem growth, cell proliferation, and regulatory hormone effects in many cases (Aguilar-Martínez et al., 2007; Martin-Trillo and Cubas, 2010). *PpTCP18* controls peach branching by positive feedback regulation of SL biosynthesis (Wang et al., 2022). In apple, *MdWUS2* can regulate branching by inhibiting *MdTCP12* expression (Li et al., 2021). The *TB1* transcription factor, also known as *FINECULM 1 (FC1)*, is a member of the *TCP* family that negatively regulates rice tillering and inhibits the subsequent growth of axillary buds (Takeda et al., 2003). The *TB1* gene is expressed at the base of the axillary bud and SAM. Its overexpression leads to a significant reduction in number of tillers, whereas in the *tb1* mutant an increased number of tillers develop (Wai and An, 2017). The transcription factor *ERF BUD ENHANCE* (*EBE*) affects cell proliferation, axillary bud growth, and branching of *Arabidopsis thaliana*. This gene encodes an *AP2/ERF* transcription factor and is highly expressed in proliferating cells (Mehrnia et al., 2013). Genes involved in plant hormone biosynthesis, transduction, and SAM formation are also associated with branching (Kurakawa et al., 2007; Ligerot et al., 2017; Zhang et al., 2018). These studies indicate that branching development is a complex process in plants.

The growth characteristics of pear rootstock have important influence on grafting effect. Sturdy seedlings and few branches make ideal stock material. In this study, the mechanism affecting pear branching formation were explored through phenotypic trait assessment, hormone content analysis and transcriptomic analysis, by using pear ‘QAU-D03’ (*Pyrus communis* L.) and less-branching mutants as materials. The results provide novel insights to improve understanding of the molecular mechanism of branch development in pear, which is of considerable importance for rootstock breeding.

## 2 Materials and methods

### 2.1 Plant materials

The original plant (OP) with more branches was a seedling progeny of ‘QAU-D03’ (*Pyrus communis* L.). The shoots of OP were exposed to gamma radiation at 50 Gy (^60^Co source, 10 Gy min^-1^) and then grafted onto one-year-old rootstocks of *Pyrus betulifolia* Bunge. Among the resulting trees, one less-branching mutant was found and named MP. One-year-old shoots of OP and MP plants were collected from the Jiaozhou Demonstration Park in Qingdao, Shandong Province, China, for tissue culture to obtain tissue-cultured plantlets. The plants were grown on MS medium with 1.0 mg·L^-1^ 6-BA, 0.1 mg·L^-1^ IBA, 30 g·L^-1^ sucrose, and 7 g·L^-1^ agar in a tissue culture room. Tissue-cultured shoots with 20 days of uniform growth were selected for rooting. Once the shoots had produced 3-4 roots of length 2-3 cm, the plantlets were transplanted to a mixture of perlite:vermiculite:peat (1:1:1, v/v/v). Samples were collected at the shoot dormancy in mid-March and bud expansion stages at the end of April, with three biological replicates per sample, for transcriptome sequencing analysis.

### 2.2 Measurement of growth traits

Annual branches from the periphery of crown of OP and MP plants, and mature leaves were randomly selected to observe leaf characteristics. Each treatment contains at least three biological replicates. The following quantitative characters were determined, including annual branch length, annual branch thickness, internode length, leaf length, leaf width and petiole length. The OP and MP of phenotypic difference was evaluated based on characteristics of pear plant.

### 2.3 Histological observation of vegetative organs

Buds and stems of MP and OP plants were sampled for conventional paraffin-embedded sectioning. The samples were fixed in formaldehyde–alcohol–acetic acid for 1 d and then dehydrated in an ethanol series (50%, 60%, 70%, 80%, and 95%) for 60 min at each step. Samples were placed in 100% ethanol and left overnight. After decoloration with dimethylbenzene, the samples were embedded in paraffin. Sections (8 μm) were cut using a rotary microtome (HI1220, Leica, Nussloch, Germany), then dewaxed, rehydrated, cleaned, stained with toluidine blue, and the coverslip mounted with neutral balata. Sections were observed and photographed using an optical microscope (RM2235, Leica, Nussloch, Germany). In addition, buds were observed by scanning electron microscopy. After manual removal of the bud scales, the buds were fixed, washed, and dehydrated as described. The material was dried by carbon dioxide critical point drying and sputter-coated with gold. The material was observed and photographed with a scanning electron microscope (JSM-7500F, JEOL, China).

### 2.4 Hormone content determination

Fresh plant stem tips material (0.2-1.0 g) was ground in an ice-cooled mortar in 10 mL of 80% (v/v) methanol extraction medium containing 1 mM butylated hydroxytoluene as an antioxidant. The extract was incubated at 4°C for 4 h and then centrifuged at 4000 rpm for 15 min at 4°C. The supernatant was filtered through Chromosep C18 columns (C18 Sep-Park Cartridge, Waters Corp., Milford, MA, USA), and prewashed with 10 mL of 100% (w/v) and 5 mL of 80% (v/v) methanol. The hormone fractions eluted with 10 mL of 100% (v/v) methanol and 10 mL ether from the columns were dried under N2 gas, dissolved in 2 mL phosphate buffer saline containing 0.1% (v/v) Tween 20 and 0.1% (w/v) gelatin (pH 7.5) for analysis by an enzyme linked immunosorbent assay (ELISA) (Bollmark et al., 1988). The ELISA was performed in a 96-well microtitration plate. Each well was coated with 100 μL coating buffer (1.5 g·L^−1^ Na_2_CO_3_, 2.93g·L^−1^ NaHCO_3_, and 0.02g·L^−1^ NaN_3_, pH 9.6) containing 0.25 μg·mL^−1^ antigens against the hormones. The coated plates were incubated for 4 h at 37°C and then kept at room temperature for 30–40 min. The plate was incubated for 3 h at 28°C for measurement of dihydrozeatin riboside (DHZR), zeatin riboside (ZR), brassinolide (BR), methyl jasmonate (JA-Me), indole-3-acetic acid (IAA), indolepyruvic acid (IPA), gibberellins (GAs), and overnight at 4°C for IAA, and then washed as described above. Color development in each well was detected using an ELISA Reader (EL310, BioTek, Winooski, VT, USA) at an optical density of A490. The contents of DHZR, ZR, BR, JA-Me, IAA, IPA, GAs, and ABA were calculated following the method of Weiler et al. (1981).

### 2.5 RNA extraction and transcriptome sequencing

Plant total RNA isolation kit (TaKaRa, Beijing, China) was used to extract total RNA from samples, following the manufacturer’s instructions, and different samples were subjected to three biological replicates. After qualification using a bioanalyzer (2,100, Agilent, United States), 1 μg of each sample was used for cDNA library construction. Four RNA sequencing libraries were constructed, in which MP1 and OP1 were the dormant shoot tips, MP2 and OP2 were the buds in expansion period. For library construction, 1 μg of RNA per sample was used as the input material. Library quality was assessed with a 2100 Bioanalyzer system (Agilent, Santa Clara, CA, USA). The clean dataset was obtained by removing reads containing the adapter sequence, poly-N, and low-quality reads from the raw data. The Q20, Q30, and GC content of the clean data were calculated. All downstream analyses used the high-quality clean data. The reference genome (*Pyrus communis* Bartlett DH Genome v2.0) and gene model annotation files were downloaded from GDR database (https://www.rosaceae.org/species/pyrus/pyrus_communis/genome_v2.0). An index of the reference genome was generated using HISAT2 v2.0.5 and paired-end clean reads were aligned to the reference genome using HISAT2 v2.0.5. FeatureCounts v1.5.0-p3 was used to count the read numbers mapped to each gene. The fragments per kilobase of exon per million mapped fragments (FPKM) value of each gene was calculated based on the length of the gene and number of reads mapped to the gene. Differential expression analysis of two conditions/groups (two biological replicates per condition) was performed using the DESeq2 R package (v1.20.0). Gene ontology (GO) enrichment analysis of differentially expressed genes (DEGs) was implemented with the clusterProfiler R package for which gene length bias was corrected. Statistical enrichment of DEGs in KEGG pathways was detected with the clusterProfiler R package.

### 2.6 Real-time quantitative PCR analysis

Total RNA was extracted from pear using the RNAprep Pure Plant Kit (Tiangen Biotech Co., Beijing, China). The cDNA was synthesized using the HiScript II 1st Strand cDNA Synthesis Kit (Vazyme Biotech Co., Nanjing, China). The Lightcycler^®^ 480 II System (Roche, Basel, Switzerland) and the ChamQ SYBR Color qPCR Master Kit (Vazyme Biotech Co.) were used to estimate relative gene expression levels under the different treatments. The reaction system (20 μL total volume) consisted of 2 μL template cDNA, 1 μL each forward and reverse primer, 10 μL Supermix, and 6 μL RNA-free water. The reaction protocol was as follows: 95°C for 5 min, then 45 cycles at 95°C for 15 s, 60°C for 30 s, and 72°C for 30 s. The Actin gene was used as an internal control. The relative expression level for each gene was calculated with the 2^−ΔΔCt^ method. Each sample analysis was repeated three times. The primers used are listed in **Supplementary Table S1**.

### 2.7 Hormone treatment

The seedlings of ‘OP’ and ‘MP’ clones were selected and subcultured with Murashige and Skoog (MS) medium (Coolaber, Coolaber Science & Technology Co.,Ltd., China). The seedlings were divided into different treatments: (i) ‘OP’ and ‘MP’ control: continued use of the MS nutrient solution; (ii) ‘OP’ and ‘MP’ treated with JA-Me: MS with 100 μmol L^-1^ Methyl jasmonate (Macklin, Shanghai Macklin Biochemical Technology Co., Ltd., China). After JA-Me treatment, observations were carried out after 10 days.

### 2.8 Statistical analysis

Statistical analysis was performed using IBM SPSS Statistics 23.0 (IBM Corporation, Armonk, NY, USA). Values are presented as the mean ± SD of at least three independent biological replicates. The significance of differences between means was analyzed with Duncan’s multiple range test or Student’s t‐test. The probability level p < 0.05 was considered to be significant.

## 3 Results

### 3.1 Comparison of OP and MP phenotypes

**Figure 1:**
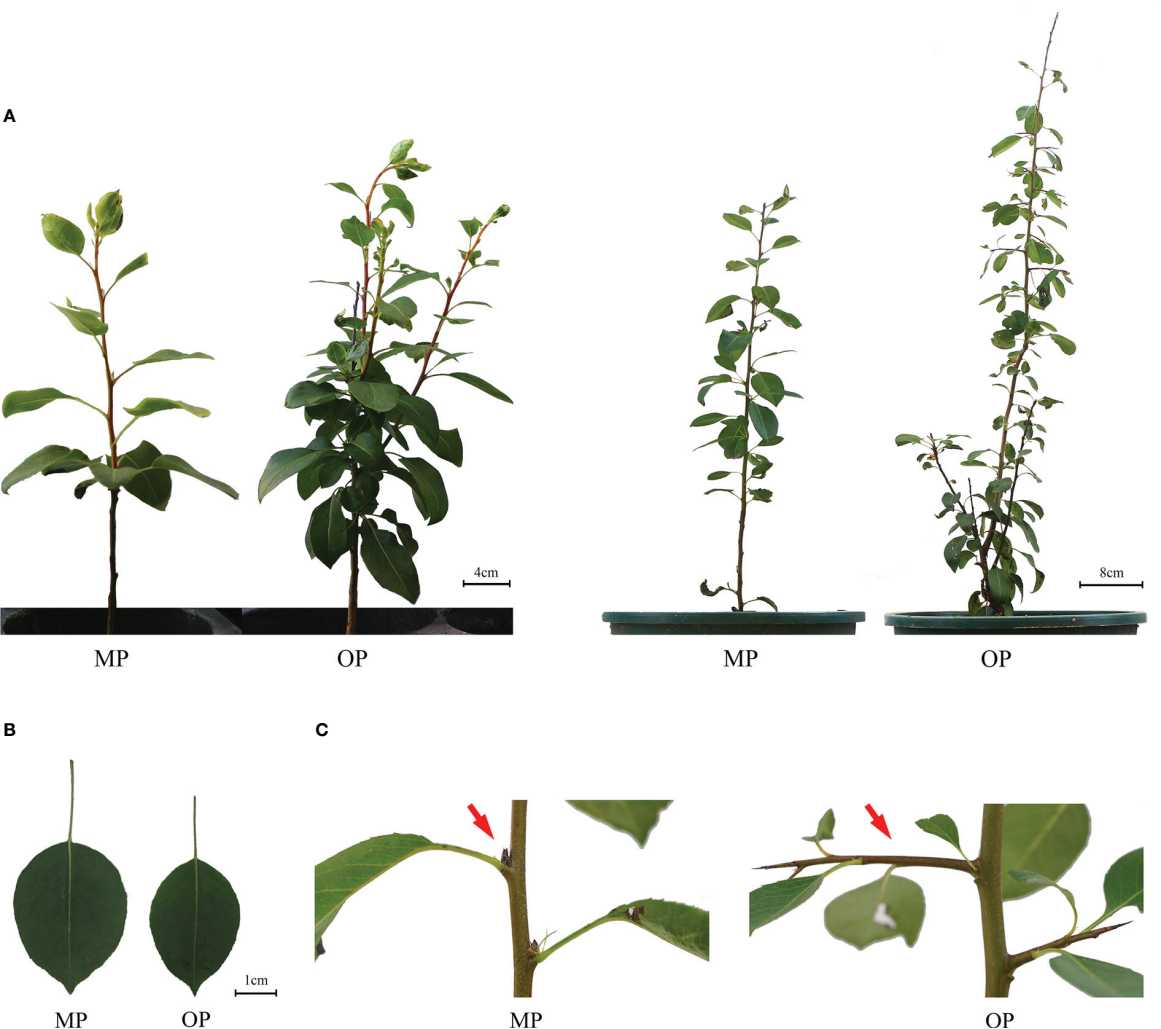
Phenotypes of the wild type (OP) and mutant (MP). **(A)** Plant phenotype of the same plant after growth for 9 months (left plant) and 12 months (right plant). **(B)** Leaf phenotype of MP and OP plants. **(C)** Axillary buds of MP and OP. The red arrow indicates transformation of the axillary meristem into a spur shoot (thorn) in OP and a dormant bud containing an undifferentiated axillary meristem in MP.

The wild type (OP) plants had more branches, whereas the mutant (MP) plants developed fewer branches and the leaves were larger (**Figure 1A**). The leaf lamina base of MP was broadly wedge-shaped or rounded, and the tip was tapered or blunt, whereas the OP leaf lamina base was mostly wedge-shaped or broadly wedge-shaped, and the tip was acute (**Figure 1B**). The mutant (MP) developed fewer branches, shorter 1-year-old shoots, and longer petiole, compared to OP, whereas no significant difference in leaf length, leaf width, leaf shape index, and annual branch thickness between OP and MP were observed (**Table 1**). With further development, the axillary meristem of OP developed into a spine-tipped spur shoot, whereas in MP axillary bud outgrowth did not occur (**Figure 1C**). All the above results confirmed that MP has the morphological characteristics of large leaves, short branches, few branches and no spines. Compared with OP, there were fewer branches in the early stage and no thorns in the leaf axils in the late stage, both of which were related to the activity of axillary meristem.

**Table 1:** Analysis of quantitative morphological characters between the mutant (MP) and wild type (OP).

Character	OP	MP
Leaf length (cm)	3.73 ± 0.43 a	4.25 ± 0.41 a
Leaf width (cm)	2.61 ± 0.30 a	3.05 ± 0.27 a
Leaf shape index	1.43 ± 0.17 a	1.41 ± 0.12 a
Petiole length (cm)	1.80 ± 0.40 a	2.97 ± 0.57 b
Internode length (cm)	1.88 ± 0.25 a	2.09 ± 0.25 b
Annual branch length (cm)	3.22 ± 0.92 a	1.36 ± 0.44 b
Annual branch thickness (cm)	0.38 ± 0.06 a	0.34 ± 0.04 a
Number of branches	2.30 ± 0.49 a	0.42 ± 0.53 b

Data are the mean ± standard error (n = 3). Different lowercase letters within a column indicate a statistically significant difference (p < 0.05, Student’s test).

### 3.2 Anatomy characteristics of the leaf, stem, and axillary bud

**Figure 2:**
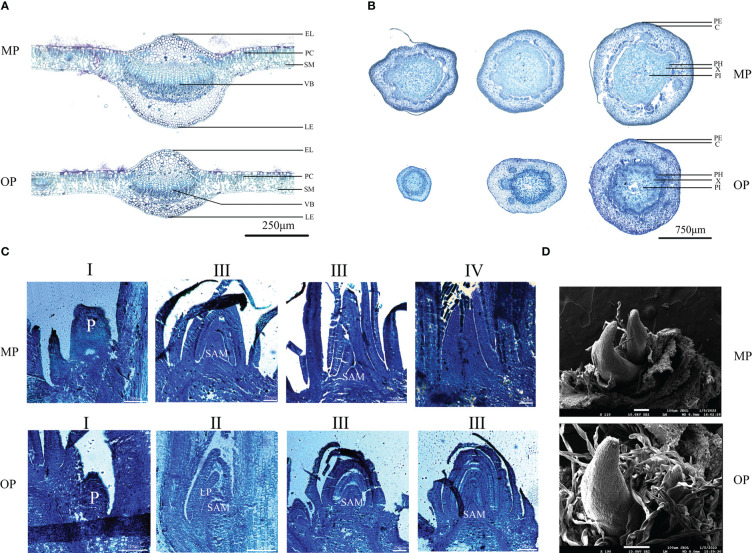
Anatomy of the vegetative organs of the wild type (OP) and mutant (MP). **(A)** Transverse sections of the leaf. EL, epithelial layer; PC, palisade mesophyll; SM, spongy mesophyll; VB, vascular bundle; LE, abaxial epidermis. **(B)** Transverse sections of the stem of the new shoots. Sections from leaf to right are from the shoot tip, mid-stem, and the shoot base, respectively. PE, periderm; C, cortex; PH, phloem; X, xylem; PI, pith. **(C)** Longitudinal sections of bud primordia at different stages of development, namely initiation preparation stage (I), initiation stage (II), formation stage (III), and maturation stage (IV). SAM, shoot apical meristem; LP, leaf primordium; P, bud primordium. **(D)** Scanning electron micrographs of dormant axillary buds.

The MP vascular bundle was larger than that of OP in the leaf, stem, and axillary buds at different development stages (**Figure 2A**; **Table 2**). The pith radius, xylem width and phloem width in the stem of new shoots of OP increased with stem development, and the phloem width changed most obviously (**Figure 2B**; **Table 3**). Axillary buds were divided into four developmental stages, namely bud primordium initiation preparation stage (I), bud primordium initiation stage (II), bud primordium formation stage (III), and bud primordium maturity stage (IV). Stage I was observed in the axillary buds of MP and OP; a group of darkly stained cells were present above the bud base, in the leaf axils of the new shoot, which represented the bud primordium (**Figure 2C**). With the further development of the bud primordium, the bud primordium entered the initiation stage (II). The growth point differentiated scale primordia from the outer to the inner, and the outermost is the scale developed from the scale primordium (LP). At the tip, the bud primordium formed the SAM and gradually differentiated into the scale leaf and bud primordium. When OP buds were in stage II, the MP buds had already entered stage III. In stage III, the bud base began to form the bud primordium, which was round and spherical, and gradually developed into a triangular form with time. In stage IV, the bud base formed a branch primordium and bracts one by one. When MP were in stage IV, the OP buds were still in stage III (**Figure 2C**). Observation of dormant axillary buds by scanning electron microscopy revealed that branch primordia and bract primordia were present in both MP and OP (**Figure 2D**). The above results showed that the bud structure of MP and OP were in different developmental stages at the same time, and the bud development of MP was faster than that of OP.

**Table 2:** Leaf anatomical characters of the mutant (MP) and wild type (OP).

Sample	VB thickness (µm)	EL thickness (µm)	LE thickness (µm)
MP	156.60 ± 0.88a	12.19 ± 0.16a	7.39 ± 0.08a
OP	96.34 ± 0.31b	11.42 ± 0.71a	7.06 ± 0.15a

VB, vascular bundle; EL, epithelial layer; LE, abaxial epidermis. Data are the mean ± standard error (n = 3). Different lowercase letters within a column indicate a statistically significant difference (p < 0.05, Student’s test).

**Table 3:** Anatomical characters of the stem in transverse section for the mutant (MP) and wild type (OP).

Sample	Position in stem	Pith radius (μm)	Xylem width (μm)	Phloem width (μm)
MP	Upper stem	243.14 ± 1.07c	29.57 ± 0.93c	51.25 ± 1.30b
	Middle stem	286.48 ± 0.92b	37.35 ± 0.62b	55.32 ± 0.96b
	Basal stem	327.18 ± 0.91a	62.84 ± 0.77a	89.05 ± 0.73a
OP	Upper stem	128.02 ± 0.97c	27.75 ± 0.51c	2.65 ± 0.63c
	Middle stem	191.51 ± 1.01b	33.04 ± 0.38b	42.25 ± 1.07b
	Basal stem	227.35 ± 0.92a	86.49 ± 0.76a	93.33 ± 1.02a

The upper stem, middle stem and basal stem were selected as 2cm below the tip of the new shoots, the middle of the new shoots and 2cm above the stem base of the new shoots respectively. Data are the mean ± standard error (n = 3). Different lowercase letters within a column indicate a statistically significant difference (p < 0.05, Duncan’s test).

### 3.3 Determination of endogenous hormone contents

**Figure 3:**
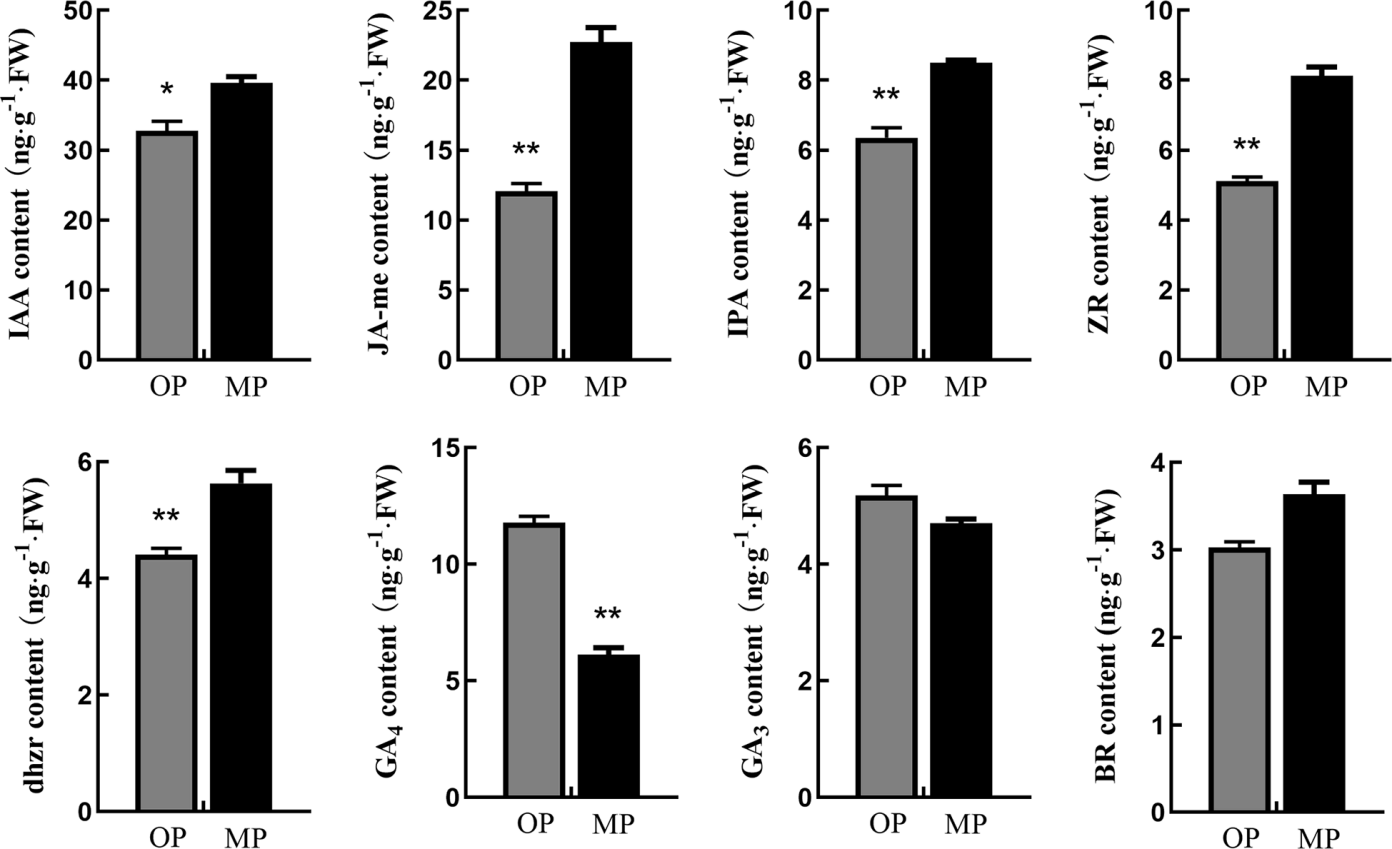
Hormone contents in stem tips of the wild type (OP) and mutant (MP). BR, brassinolide; DHZR, dihydrozeatin riboside; GA3 and GA4, gibberellins; IAA, indole-3-acetic acid; IPA, indolepyruvic acid; JA-Me, methyl jasmonate; ZR, zeatin riboside. Error bars indicate the standard error. *p < 0.05, **p < 0.01 (Duncan’s test).

Endogenous hormone analysis revealed that the content of GA4 in OP was significantly higher than that in MP, whereas the contents of auxin (IAA), methyl jasmonate (JA-Me), indolepro pionic acid (IPA), trans-zeatin-riboside (ZR), brassinolide (BR), and dihydrozeatin-riboside (DHZR) in MP were higher than those in OP. The difference in JA-Me content between OP and MP was most marked (**Figure 3**). Based on these results and the following transcriptome analysis, we speculate that jasmonic acid may play a role in regulating pear branching.

### 3.4 Gene ontology and KEGG pathway analysis of DEGs

**Figure 4:**
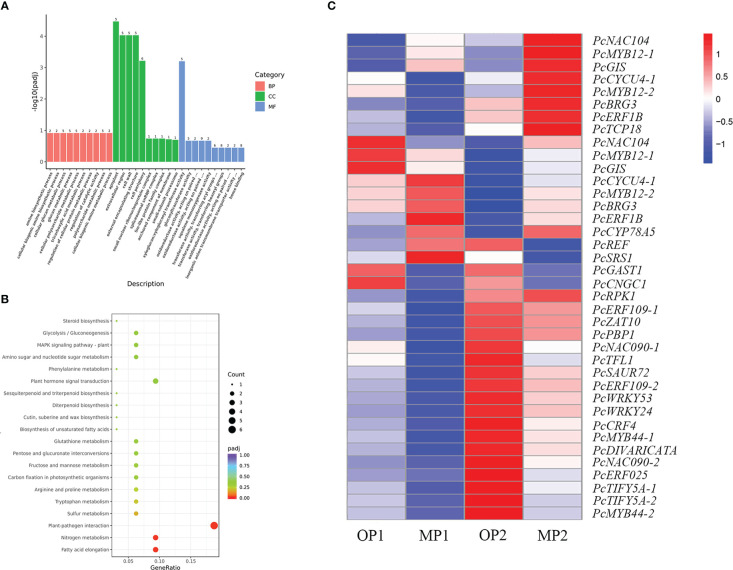
Functional annotation of differentially expressed genes (DEGs) in the mutant (MP) and wild type (OP). **(A)** DEGs enriched among the top 30 genes ontology terms. The annotated items are divided into three categories: cellular components(CC), molecular functions(MF), and biological processes(BP). **(B)** DEGs enriched among the top 20 KEGG pathways. **(C)** DEGs involved in cell division, differentiation, and shoot apical meristem activity, in which MP1 and OP1 were the shoot dormancy, MP2 and OP2 were the bud expanding stage. FPKM values are log_2_-based.

The MP and OP shoot tips were sampled for transcriptome analysis. A total of 334 DEGs were common to the MP1 vs. OP1 and MP2 vs. OP2 comparison groups. Enrichment analysis of the GO terms indicated that the DEGs were mainly enriched in the apoplast in the biological process category, extracellular regions in the cellular component category, and xyloglucosyl transferase activity in the molecular function category (**Figure 4A**). The enrichment of KEGG pathways indicated that the DEGs were mainly enriched in the pathways of plant-pathogen interaction, fatty acid elongation, and nitrogen metabolism (**Figure 4B**). Thirty-eight of the 334 DEGs were associated with plant growth, cell division and differentiation, and SAM activity. Among these 38 DEGs, 23 genes encoded transcription factors, including members of the *AP2/ERF*, *P450/CYP*, *MYB*, *WRKY*, *TCP*, and *NAC* families (**Figure 4C**).

### 3.5 DEGs involved in plant hormone signaling pathways

**Figure 5:**
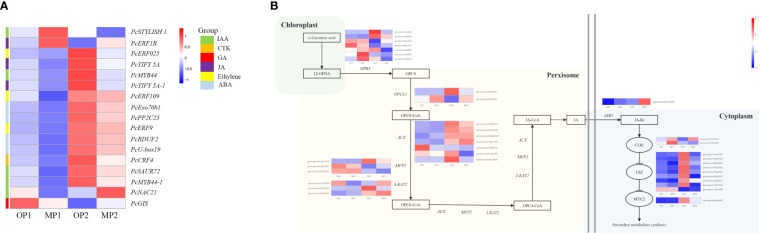
Differentially expressed genes involved in plant hormone signaling pathways and in jasmonate biosynthetic and signal transduction pathways. **(A)** Hormone-related DEGs, in which MP1 and OP1 were the shoot dormancy, MP2 and OP2 were the bud expanding stage. **(B)** Pathway constructed based on KEGG pathways and the literature. The enzymes and intermediates are indicated as follows: OPR3, OPDA reductase; ACX, acyl-coenzyme A oxidase; MFP2, peroxisomal fatty acid beta-oxidation multifunctional protein; 3-KAT2, 3-ketoacyl-CoA thiolase 2; JAR1, jasmonate resistant 1; OPC8, 8-(3-oxo-2-(pent-2-enyl)cyclopentyl) octanoic acid; JA, jasmonic acid; JA-Ile, jasmonoyl-L-isoleucine. FPKM values are log_2_-based.

The hormone-related DEGs were screened and the changes in their expression in the two control groups were analyzed. Among these DEGs, most genes were upregulated in OP2, including IAA, JA-Me, Ethylene, ABA, CTK. Genes involved in the gibberellin signaling pathway were highly expressed in OP1 (**Figure 5A**). With regard to the JA biosynthesis and regulation pathways, 35 detected genes were associated with the octadecane synthesis pathway starting from α-linolenic acid, including genes encoding plant dienoic acid reductase (OPR3), acetyl coenzyme A oxidase (ACX), peroxisome fatty acid oxidized multifunctional protein (MFP2), and ketoethyl coenzyme A thiolase (3-KAT2), which are all involved in JA biosynthesis in the peroxisome. *COI1-related* genes were highly expressed in MP, whereas *JAZ* protein-related genes and *MYC-related* transcription factors were highly expressed in OP (**Figure 5B**). In OP and MP, the expressions of *COI1-related* genes and *JAZ* protein-related genes showed regular changes. We speculated that the phenomenon of plant branching might be related to *COI1* and *JAZ*.

### 3.6 Jasmonic acid negatively regulates branching formation

**Figure 6:**
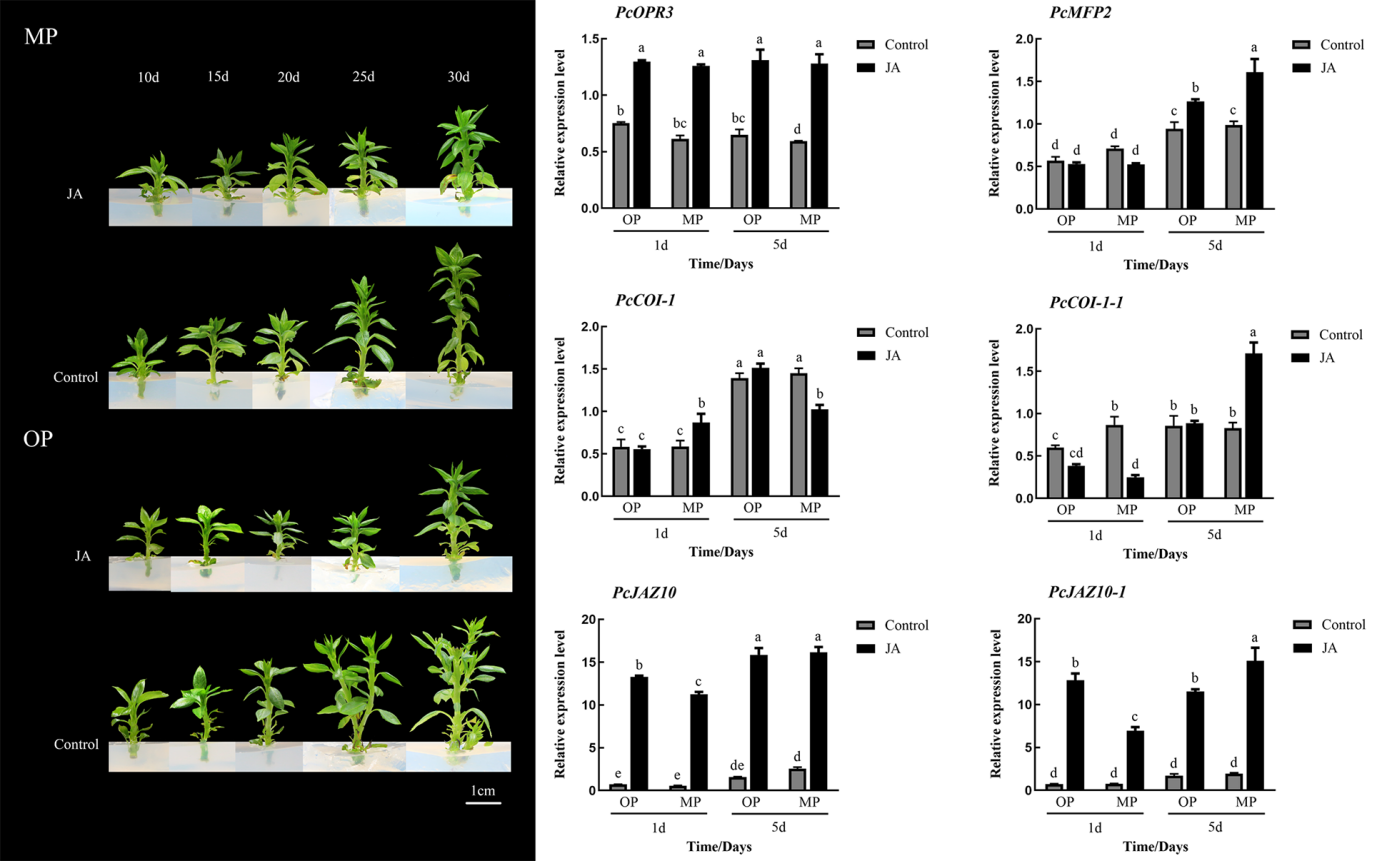
Effects of exogenous methyl jasmonate (JA) on plant phenotype and relative expression of six genes associated with the jasmonate signaling pathway at different stages of shoot development. Different lowercase letters above bars within a graph indicate a statistically significant difference (p < 0.05, Duncan’s multiple range test).

Exogenous application of 100 μmol·L^−1^ JA-Me did not notably affect the branching of MP plants, whereas the branching of OP plants was visibly inhibited, in contrast to the control but consistent with the phenotype of MP plants. Expression analysis showed that the expression of *PcOPR3* was significantly up-regulated after JA-Me treatment. In OP plants, *PcCOI1* was not significantly increased in response to JA-Me treatment, whereas *PcCOI1* was significantly upregulated in MP. *PcJAZ1* was significantly upregulated in OP and MP in response to JA-Me treatment, but the extent of upregulation in OP was less than that in MP (**Figure 6**).

## 4 Discussion

Pear is an economically important deciduous fruit tree. However, pear has a long growth cycle and complex genetic background, which is usually propagated by grafting. The growth characteristics of rootstocks play an important role in the outcome of grafting. Strong seedlings with few branches are ideal rootstock materials. In the present study, compared with OP, MP developed fewer branches, but the regulatory mechanism for these phenotypic changes remains unclear. Exploration of the regulation of pear branching through comparative analysis is important for the breeding of seedlings suitable for rootstocks.

Morphological assessment is the most common and direct method of identifying bud mutations. In this study, the morphology of OP and MP differed significantly. The MP plants had the phenotypic characteristics of few branches and large leaves, whereas OP had the contrasting traits of many branches and small leaves. Compared with MP, OP developed more branches at an early stage and the leaf axillary buds produced spiny spur shoots. Similar results have been reported in other species, such as birch and larch (Han et al., 2019; Cai et al., 2021). In addition, the vascular bundles and abaxial epidermal cells of MP leaves were larger than those of OP, and the areal proportion of the pith, xylem, and phloem in new shoots of MP from the base to the tip was greater than that of OP, and almost no conducting tissue had differentiated at the shoot tip of OP. These results indicated that MP had a greater capacity for nutrient and water transport than OP, which may be one reason why MP had stronger main branches and fewer branches. The bud structure and development period of MP and OP were different; bud development was more rapid in MP than in OP, thus the bud growth patterns were indicated to differ between OP and MP.

Cell division, expansion, and differentiation affect the basic processes of plant organ growth and development, and ultimately affect plant phenotype (Sugiyama, 2005; Yang et al., 2015). Plant morphogenesis is closely associated with genes involved in cell division, expansion, and differentiation (Lopez-Hernandez et al., 2020). The diversity of plant morphology is largely affected by SAM activity (Sussex and Kerk, 2001). In the current study, 38 DEGs associated with cell division, differentiation, and SAM activity were identified, including 23 transcription factors belonging to the AP2/ERF, P450/CYP, TCP, and NAC families. Transcription factors play a vital role in plant development and regulation of gene expression, forming a complex gene regulatory network (Muiño et al., 2016; Yang et al., 2018). Most of the 23 transcription factors were highly expressed in OP and participate in the regulation of mutant branching traits. The *TCP* transcription factors are involved in the growth of lateral meristems, cell proliferation, and regulation of hormones (Aguilar-Martínez et al., 2007; Martin-Trillo and Cubas, 2010). In citrus, the *TCP* transcription factors *THORN IDENTITY 1 (TI1)* and *TI2* are essential to preclude the proliferation of meristems and simultaneously produce thorns (Zhang et al., 2020; Zhang et al., 2021). In the present study, the expression of *PcTCP18* in OP was higher than that in MP at an early stage of development, whereas expression was higher in MP than in OP at an advanced stage of development. The gene *PcTCP18* has high homology with the citrus *TI1* gene. In peach, *PpTCP18* can reduce secondary branches through brassinolide pathway (Wang et al., 2022). Based on these results, we speculated that *PcTCP18* may be a candidate gene responsible for reducing the branching of MP at an early stage of development.

Plant hormones can regulate plant development processes, such as cell division, bud development, branch branching, and senescence (Heyl et al., 2007). Genes associated with hormone metabolism and signal transduction play an important role in regulating plant and organ size (Guo et al., 2010). In the present study, MP and OP were revealed to differ significantly in hormone contents. The IAA and cytokinin contents in MP were significantly higher than those in OP. Furthermore, the expression level cytokinin-related genes in OP were also higher than that in MP, indicating that cytokinins played an important role in plant branching. This result was consistent with a previous report that cytokinin promotes plant branching, tillering development, and lateral bud growth (Foo et al., 2007). Gibberellins play an important regulatory role in plant growth and development, and can control vertical growth and branching (Arend et al., 2009). In the current study, the expression of DEGs associated with GAs was higher in OP. Similarly, the GA_4_ content in OP was higher than that in MP. Gibberellin had been proved to have the effect of inhibiting branching, especially the development of axillary buds was closely related to the content of gibberellin (Martinez-Bello et al., 2015; Tan et al., 2018). This also showed that gibberellin played an important role in the branch development of pear trees. In addition, the JA content in MP was significantly higher than that in OP. Jasmonic acid has diverse roles in regulating developmental processes such as seed germination, root development, and senescence (Jin and Zhu, 2017). Exogenous JA-Me treatment and *JAZ* mutants all can cause the phenotype of branch reduction (Hong et al., 2020). Auxin can also cause changes in plant branching through jasmonic acid (Wang et al., 2013). In our study, 35 genes related to jasmonate biosynthesis and signal transduction were differentially expressed. Among them, in the JA signaling pathway, *PcCOI1-related* genes were highly expressed in both stages of shoot development in MP, whereas *PcJAZ*s were expressed at a lower level in MP, indicating that Jasmonate signal transduction is involved in the formation of branching. Application of exogenous JA-Me did not notably change the MP branching phenotype, whereas OP treated with exogenous JA-Me showed a decrease in frequency of lateral branching, resulting in a branching phenotype similar to that of MP. The expression of *PcOPR3* was significantly up-regulated after JA-Me treatment. The downstream JA regulatory gene *PcCOI1* was not highly expressed in OP, but was significantly upregulated in MP plants, in response to JA-Me treatment. *JAZ* negatively regulates the downstream regulatory pathway of JA. In response to JA-Me treatment, the expression level of *PcJAZ* was significantly increased. So far, there were few reports about the effect of jasmonic acid on the branches, but previous studies had confirmed that JAZ could promote growth and inhibit aging and COI1 also participated in plant growth and development (Huang et al., 2017; Oblessuc et al., 2020; ). Therefore, we concluded that JA caused the decrease of plant branch development due to the inhibition of jasmonate on growth, and the jasmonate regulatory gene in OP was not sensitive to jasmonate.

In conclusion, through observation of the phenotypic characters of MP and OP, the mutation in MP results in reduced branching, large leaves, and slower development of bud primordia. The contents of JA, IAA, IPA, and ZR were significantly higher in MP than in OP, especially that of JA. The wild type showed a little-branching phenotype after treatment with exogenous JA-Me. The expression levels of JA synthesis regulatory genes in MP were increased and these genes were responsive to application of exogenous JA-Me.

## Data availability statement

The data presented in the study are deposited in the National Center for Biotechnology Information (NCBI) BioProject database repository, accession number PRJNA916958.

## Author contributions

YC and CL conducted the experiment, and YC prepared the manuscript. ZQ, SZ, and JL provided important technical help in transcriptional analysis, YY, RW, and CM gave useful guidance and valuable discussion. JY, ZC, and JS provided the plant materials and important technical help in histological Observation. DL conceived the idea and provided financial support. All authors contributed to the article and approved the submitted version.
