# Supplementary material for: Jasmonic acid negatively regulates branch growth in pear
Source: Front Plant Sci. 2023 Feb 7;14:1105521. doi: 10.3389/fpls.2023.1105521 (PMC9941643; doi:10.3389/fpls.2023.1105521)
Supplement: Supplementary file 1 [file Table_1.docx]

**SUPPLEMENTARY TABLE S1 |** Primers used in the study

| **Gene** | **Primer name** | **Primer sequence (5′–3′)** |
| --- | --- | --- |
| *PcActin* | F | ACAGTGTCTGGATTGGAGGGTC |
|  | R | CAT TTG GAG AAC TCA GAA GCAC |
| *PcOPR3* | F | TGGACGTAGTTCACGCCAAA |
|  | R | TCTCCACCTCTTTGAAATGGGA |
| *PcMFP2* | F | GAAAACCCCAGTGGTTGTTGG |
|  | R | AACATCGGCACCACGTTCA |
| *PcCOI-1* | F | CTCGACAAGTGCTCAGGGTT |
|  | R | CCAATTGCCGTCCTTCTCCT |
| *PcCOI-1-1* | F | TCTCTGTGATTTTCGCCTTTTCTG |
|  | R | CTGCTGGCATCCCCTCAAAA |
| *PcJAZ10* | F | AGAGCCTCCTGGAAACAACG |
|  | R | AGCTTTGCGGGAGTTTCCTT |
| *PcJAZ10-1* | F | AGCCGCTGACGATTTTCTACA |
|  | R | CGGCTACCGATGCCGATG |
